# Why Homoscleromorph Sponges Have Ciliated Epithelia: Evidence for an Ancestral Role in Mucociliary Driven Particle Flux

**DOI:** 10.1002/jez.b.23324

**Published:** 2025-08-22

**Authors:** Veronica L. Price, Anudi Nanayakkara, Andrea Pasini, Elsa Bazellières, Amelie Vernale, Caroline Rocher, Carole Borchiellini, Andre Le Bivic, Emmanuelle Renard, Sally P. Leys

**Affiliations:** ^1^ Department of Biological Sciences University of Alberta Edmonton Canada; ^2^ Aix Marseille Univ, CNRS, Institute of Developmental Biology of Marseille (IBDM UMR 7288) Marseille France; ^3^ Aix Marseille Univ, CNRS, IRD, IMBE UMR 7263, Avignon Université, Institut Méditerranéen de Biodiversité et d'Ecologie marine et continentale, Station Marine d'Endoume Marseille France

**Keywords:** cilia, epithelia, high speed imaging, homoscleromorph, mucociliary, Porifera, sponge

## Abstract

Cilia are found on the epithelia of almost all metazoans, so their absence from the epithelia of all but one class of Porifera is puzzling. Homoscleromorph sponges possess ciliated epithelia, but their function and evolutionary history within Porifera are unclear. We compared the ciliary beat frequencies (CBFs) of cilia on outer epithelia of the homoscleromorph sponge *Oscarella* sp. with those of other animals to suggest possible functions for the cilia. Settled Stage 4 buds, or juveniles, were found to have a higher CBF than free‐moving Stage 1 buds, and CBF was within the range of cilia that function in mucus transport in other aquatic invertebrates. Scanning Electron Microscopy (SEM) images of buds fixed with ruthenium red to detect the presence of mucus showed that mucus was associated with the cilia of the exopinacoderm and both SEM and immunofluorescence images revealed fields of homogeneously oriented cilia. Confocal imaging of fluorescent beads also showed that cilia beat in the same direction. Movement of beads was reduced by nocodazole treatment indicating that the movement of particles over the surface was caused by ciliary beat. These results suggest that cilia on the epithelia of Homoscleromorph sponges are involved in mucociliary‐driven particle flux, and may be used to clean the surface using mucus.

## Graphical abstract

Epithelia are typically ciliated, except in sponges. Of all Porifera only Homoscleromorphs have motile cilia on their epithelia. Our data highlight the presence of cilia and mucociliary particle transport as a common feature of metazoa and a secondary loss in other sponge lineages.

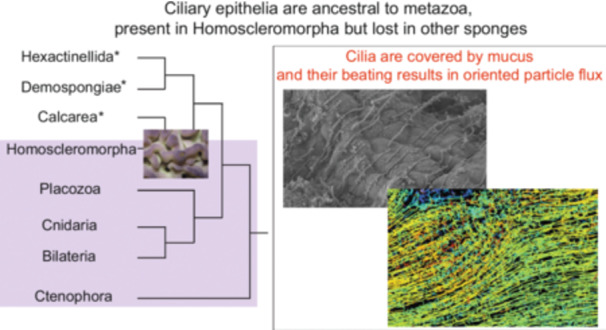

**Abbreviations**
CBF: ciliary beat frequency
cl: ciliated larva
mu: mucus
PBS: phosphate buffered saline
sc: sensory cilia
SEM: scanning electron microscopy

## 1 Introduction

Cilia and flagella are tubulin‐based, hair‐like organelles that arise from the surface of cells. Their diverse functions include locomotion, mucus transport, egg transport, feeding, sensation, and signalling (Mitchison and Valente 2017; Gilpin et al. 2020). Dysfunctions of cilia or flagella are the cause of numerous diseases known as ciliopathies (Reiter and Leroux 2017). Whereas the absence of cilia from the epidermis of Ecdysozoa is fully explained by the presence of a cuticle, the absence of cilia on the epithelia of three out of four sponge classes is puzzling. Unusually, only one class of sponges, Homoscleromorpha, possesses ciliated epithelia (Ereskovsky et al. 2009; Leys and Hill 2012; Ereskovsky et al. 2014; Renard, Rocher, et al. 2021; Rocher et al. 2024). The function of these cilia, as well as the evolutionary history of this characteristic trait, are unknown.

The terms cilia and flagella are often used interchangeably, but although they describe organelles with the same morphological structure (Sleigh 1974), they differ in how they beat and consequently whether they deflect water along or away from the surface of the cell. Flagella beat in sinusoidal waves drawing water to their base and up along the organelle perpendicular to the cell, while cilia beat in forward (effective) and backward (recovery) strokes, moving fluids parallel to the surface of the cell (Sleigh 1974); some cilia are nonmotile and considered to have a sensory function (Singla and Reiter 2006). Cilia are common throughout Metazoa, while flagella are seen only in sperm of Metazoa, choanocytes of Porifera, and flame cells of Plathyhelminthes (Brusca et al. 2016). Cilia come in a range of forms which are related to function. Cells may be monociliated or multiciliated (Brooks and Wallingford 2014) and in some animals cilia are grouped together under one membrane to form macrocilia, as in Ctenophora (Tamm 2014). Cilia also form many of the sensory cells found in Metazoa, from apical organs of larvae to a myriad of types of photoreceptors (Eakin 1982; Bezares‐Calderón et al. 2020).

Among the non‐bilaterian phyla, Placozoa and Cnidaria both have mono‐ciliated epithelia (Rassat and Ruthmann 1979; Nielsen 1987), while Ctenophora have a vast range of ciliated epithelia including macrocilia used as teeth, and others which form comb‐plates used for locomotion (Horridge 1964). In Porifera (sponges), larvae are typically fully ciliated, and generally monociliated, although the larvae of two species of deep‐sea sponges (*Lycopodina occidentalis*, Demospongiae, and *Oopsacas minuta*, Hexactinellida) have multiciliated cells (Boury‐Esnault et al. 1999; Leys et al. 2006). However, in almost all Porifera, cilia are either shed from the larval cells at metamorphosis or they are resorbed into the larval cells which then transdifferentiate into the flagellated cells that form the choanocyte chambers (Leys and Degnan 2002; Ereskovsky et al. 2007; Ereskovsky et al. 2010; Sogabe et al. 2016), but they are never found on the exopinacoderm epithelia of the adult sponge except in homoscleromorphs.

Cilia are such essential organelles on metazoan epithelia that their absence from the epithelia of three out of the four sponge classes is puzzling. Indeed, only sponges in the small class Homoscleromorpha (~140 species; de Voogd et al. 2024) retain a fully ciliated epithelium (exo‐ and endopinacoderm) as juveniles and adults (Ereskovsky et al. 2007; Ereskovsky et al. 2009; Rocher et al. 2024). That is not to say there are no cilia on some cells in the other classes of Porifera: short, 4–6 micron long nonmotile cilia have been found in the oscula of sponges from classes Demospongiae and Hexactinellida and motile cilia are found on cells that form the apopyle, which is the exit to choanocyte chambers (Hammel and Nickel 2014; Ludeman et al. 2014; Leys et al. 2025). Curiously, so far, no cilia have been found on the epithelia of Calcarea. This raises the question as to whether the ancestral condition in Porifera was a ciliated pinacoderm, and if so, why many sponges have lost ciliated epithelia.

While the evolutionary relationships among non‐bilaterians are still unresolved (Dunn et al. 2015; Redmond and McLysaght 2021; Juravel et al. 2023; Schultz et al. 2023), Poriferan relationships are well‐established with Demospongiae and Hexactinellida forming a sister group to Homoscleromorpha and Calcarea (reviewed in, Wörheide et al. 2012). Homoscleromorphs have a number of traits that are distinct from the other classes of Porifera including the presence of a clear basement membrane and adhaerens‐like junctions (seen also in Calcarea) between epithelial cells (Renard, Le Bivic, et al. 2021).

The presence of a ciliated epithelium in homoscleromorph sponges, and its absence in epithelia of other adult Porifera is a puzzle that has yet to be addressed by any study. Here we explored the function of the cilia on the external epithelia of homoscleromorph sponges of the *Oscarella lobularis* species complex, referred to here as *Oscarella* sp., marine sponges found in shallow waters of the Mediterranean Sea (Ereskovsky et al. 2009; Guiollot et al. 2024). These sponges have both sexual and asexual reproduction. Asexual reproduction produces tiny spherical ciliated buds (Ereskovsky and Tokina 2007; Vernale et al. 2021). Not only are these easy to study in the lab (Vernale et al. 2021; Rocher et al. 2024), but because of their small size it is possible to image the beat of cilia on their epithelia in living specimens by light microscopy.

We considered that cilia on the exopinacoderm of *Oscarella* sp. could be involved in mucus transport for cleaning the surface, in enhancing flow into the incurrent canals, or in food capture. To better understand which of these possible functions might take place, we compared the ciliary beat frequency (CBF) and patterns of beat of cilia on the surface of buds with those of animals that use cilia for mucus transport or food capture (e.g., clam gill) and others where cilia rather have a predicted sensory function (e.g., anemone tentacle, clam gill, and sponge apopyle cilia). We also carried out a meta‐analysis of ciliary beat patterns and characteristics of ciliated epithelia across Metazoa to better understand the function of cilia on the epithelia of homoscleromorph sponges.

## 2 Materials and Methods

### 2.1 A Note on the Taxonomy of *Oscarella lobularis*

*Oscarella lobularis*, which exists as a species complex (Guiollot et al. 2024) in the Mediterranean Sea, lacks a spicule skeleton that is typically used for identifying sponge species. Although the specimens we collected came from the same sites as those previously referred to as *O. lobularis*, because they are now known to form a species complex, we therefore refer to the individuals used in this study as *Oscarella* sp.

### 2.2 Video Imaging of Tissues

We used a high speed camera (MegaSpeed 5, Canadian Photonics, Minnedosa, Canada) to image cilia beating on buds of *Oscarella* sp. (Porifera, Homoscleromorpha), on the tentacles from the anemone *Nematostella vectensis* (Cnidaria, Anthozoa), on the gill of the clam *Venerupis philippinarum* (Mollusca, Bivalvia), in the choanocyte chambers of the sponge *Ephydatia muelleri* (Porifera, Demospongiae), in the branchial basket of the tunicate *Corella inflata* (Urochordata, Ascidiacea), on the lophophore of a brachiopod *Terebratalia transversa* (Brachiopoda, Rhynconellata), and on the lower epithelium of the placozoan *Trichoplax H2* (Placozoa). Two individuals of *Oscarella* sp. were collected from the Bay of Marseille, France. *Trichoplax H2* were obtained from a population kept in culture at the Marseille Institute for Developmental Biology. *V. philippinarum* and *C. inflata* were obtained from the docks of the Bamfield Marine Sciences Center, BC, Canada, and *N. vectensis* was originally obtained from A. Reitzel and maintained as a laboratory culture for many years at the University of Alberta, Canada. *E. muelleri* was cultured in the lab from gemmules collected from O'Connor Lake, Vancouver Island. Since only one individual of the brachiopod *Terebratalia transversa* was obtained from the invertebrate zoology labs at the University of Alberta, only qualitative observations were made on this organism and the beating rates were not included in comparative analyses.

*Oscarella* sp. buds were obtained as described in Rocher et al. (Renard, Rocher, et al. 2021; Rocher 2024). Briefly, adults were first cut into 1 cm^3^ pieces using a sterile scalpel, and each fragment was placed in a well of a six‐well cell culture plate containing natural sea water and maintained at 17°C. Buds released from the adult fragments were transferred into Petri dishes containing 8 mL natural seawater and maintained at 17°C. Buds were collected at developmental Stage 1 (newly formed), 2 (within a week), and Stage 4 (1‐month‐old juveniles). Buds at developmental Stage 1 float; therefore, the beat of cilia on their external epithelia move them very slowly, without any uniform direction, through the water. In contrast, buds at developmental Stage 4 adhere to a substrate and are therefore immobile, hence called ‘juvenile’ sponges (Rocher et al. 2024). Live buds were placed in MatTek coverslip bottom dishes, and filmed on a Colibri Zeiss inverted microscope, using a MegaSpeed5 camera.

*Trichoplax H2* were filmed in MatTek coverslip bottom dishes on a Colibri Zeiss inverted microscope as described above, using a MegaSpeed5 camera. *E. muelleri* sponges were hatched from gemmules onto MatTek coverslip bottom dishes (Leys et al. 2019). Choanocyte chambers in transparent regions of Stage 5 sponge juveniles were imaged using a Zeiss Axiovert 2 M microscope and a MegaSpeed5 camera. The tentacle of *N. vectensis*, the gill of *V. philippinarum*, the lophophore tentacles of *T. transversa* and brachial baskets of *C. inflata* were all imaged in MatTek dishes on a Zeiss Axiovert 2 M using the MegaSpeed5 camera. All image sequences were captured at 100 and 500 frames per second.

Image sequences were processed in ImageJ Fiji (Schindelin et al. 2012) to enhance contrast, and scale bars and time stamps were added. To reduce file sizes for image analysis, each image sequence was cropped to retain only the section of interest. Image sequences were analyzed in MegaSpeed's AVI Player. By advancing images frame by frame a motile cilium was selected that was both in focus and easily distinguishable from other nearby cilia. The time taken for the cilium to return to a chosen starting position was recorded as the time for one beat; a minimum of 10 beats were recorded for each individual cilium. Data were exported to MS Excel (2016) to calculate average time per beat (ciliary beat frequency, CBF) in Hertz (Hz) (Table 1).

**Table 1:** Number of cilia (*n*) used to obtain average CBF for each organism and the number of videos from which they were obtained.

Species	No. of videos	No. of cilia counted	No. of individuals
*Oscarella* sp. Stage 1	3	25	3
*Oscarella* sp. Stage 4	12	70	3
*Nematostella vectensis*	3	6	2
*Venerupis phillipinarum*	13	47	3
*Trichoplax H2*	10	29	3
*Ephydatia muelleri*	12	21	3
*Terebratalia transversa*	17	56	1
*Corella inflata*	5	11	3

### 2.3 Fluorescent Imaging of Ciliary Beat

Buds of *Oscarella* sp. were immobilized in a glass‐bottomed Petri dish by placing a coverslip on top of them. Red fluorescent beads (200 nm diameter) were added to the surrounding seawater and imaged using an AxioObserver Z1 equipped with a Colibri LED system in both control and nocodazole‐treated (1 mM) samples. Images were acquired every 70 ms using a 40× water immersion objective. The tracks of the beads were automatically segmented using the TrackMate plugin on Fiji software (Tinevez et al. 2017). As the bead diameter was less than 5 pixels, the DoG detector was used. This detector uses the difference of Gaussians approach to approximate a LoG filter by two Gaussians and it is thus optimal for spot sizes less than 5 pixels. The simple LAP tracker algorithm was then chosen to track the path of the beads as these particles do not divide nor merge and undergo Brownian motion. The path of each bead was visualized according to its average speed.

### 2.4 Fluorescence and Scanning Electron Microscopy

To better characterize the cilia from each organism, tissues were fixed for immunofluorescence and Scanning Electron Microscopy (SEM). For fluorescence *Oscarella* sp. buds were fixed as described previously (Rocher et al. 2024). Briefly, they were placed in 3% paraformaldehyde in phosphate‐buffered saline (PBS) at 4°C, rinsed three times in PBS for 15 min each, blocked in 2% blocking reagent (Roche) with 0.1% saponin and stained with anti‐acetylated α‐tubulin (Sigma, 1:500) at 4°C overnight. Buds were rinsed three times in PBS and incubated for 1 h in secondary antibody, Alexa Fluor 488 anti‐mouse (Life Technologies). Samples were rinsed as above and counterstained in phalloidin (Alexa Fluor 568 or 594, ThermoFisher) and DAPI (2 mg mL^−1^ stock, diluted to 1:500). Samples were mounted in ProLong Diamond mounting medium (Invitrogen) and viewed and imaged on a Zeiss LSM 780 (Borchiellini et al. 2021; Rocher et al. 2024).

For SEM, *Oscarella* sp. buds, whole *N. vectensis*, and portions of the gill of *V. philippinarum* were fixed in a cocktail containing 2% glutaraldehyde and 1% osmium tetroxide in 0.45 M sodium acetate buffer at pH 6.4, with or without 1 mg mL^−1^ ruthenium red in the final fixative (Leys 1995). Ruthenium red stains polysaccharides and has been shown to highlight mucus on a range of tissues (Luft 1971; Lavrov et al. 2022). Some individuals of *N. vectensis* were anesthetized in 0.35 M MgCl_2_ mixed 1:1 with one‐third concentration (11 g L^−1^) seawater, for 30 min before fixation, while other anemones, portions of gills and tentacles were dropped directly into fixatives. Tissues were rinsed in distilled water three times, dehydrated to 100% ethanol, and fractured by placing the vials in liquid nitrogen. After fracturing, pieces were thawed to room temperature, critical point dried in a Bal‐Tec CPD 030 critical point dryer, mounted on aluminum stubs, and viewed in a Zeiss Sigma 300 Field Emission SEM at the University of Alberta.

### 2.5 Meta‐Analysis

A meta‐analysis of the literature was carried out to gather rates of ciliary beat from different tissues in a diversity of animals for comparison with ciliary beat rates from *Oscarella* sp.

### 2.6 Statistical Analysis

A single‐factor ANOVA test and Tukey's HSD test were used to compare CBF across animals. All graphing and statistical analysis were carried out using GraphPad Prism version 9.5.1. Results were determined to be significant at *p* < 0.05.

## 3 Results

### 3.1 Overview of the Ciliary Pinacoderm Epithelium

**Figure 1:**
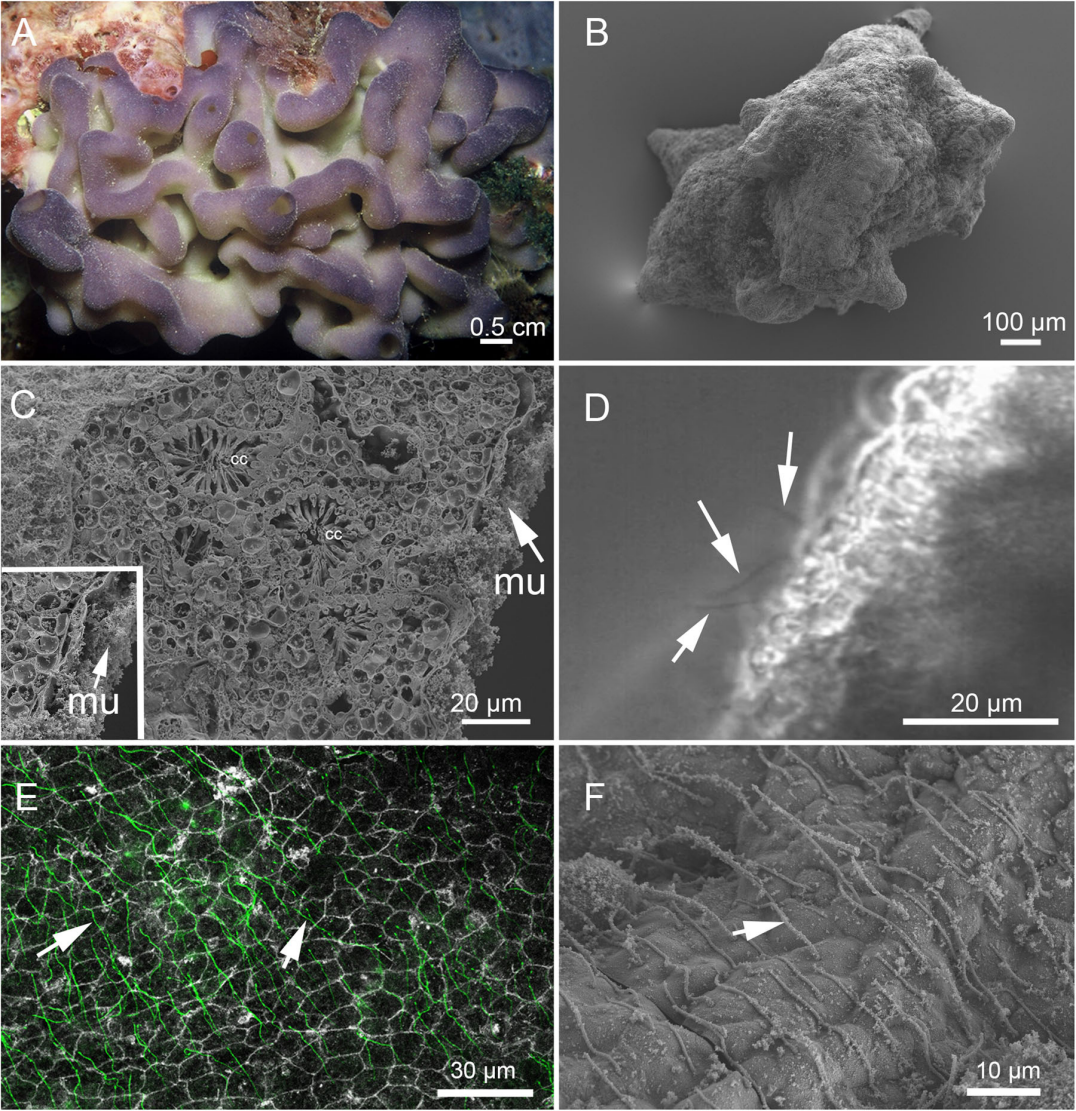
Cilia on the marine sponge *Oscarella* sp. (A) An adult specimen of *Oscarella* sp. (B) SEM image of a bud. (C) SEM image of a fracture through a Stage 4 bud showing the ciliated epithelium on either side. The bud was fixed with ruthenium red to reveal mucus (mu, inset). Cc, choanocyte chambers. (D) Image of the ciliated epithelium of a Stage 4 bud taken with a MegaSpeed 5 camera. Arrows indicate cilia at different stages of beating. (E) Cilia (arrows) on the surface of a bud labelled with anti‐tubulin (cilia, green, anti‐tubulin; cell boundaries, white, phalloidin). (F) SEM of cilia on the surface of a Stage 4 bud shows cells are monociliate and cilia (arrow) are oriented in the same direction.

The individuals from which buds were derived had a purple‐pink colour at the surface that was paler near their attachment site (Figure 1A). Young buds were irregular spheres with stubby knobs projecting from all sides (Figure 1B). In section, buds were hollow balls, and the walls of the balls had all the components of a functioning sponge: outer and inner epithelia (exo‐ and endopinacoderm respectively), choanocyte chambers with small canals leading towards and away from them, and a collagenous mesohyl filled with a few cells and a lot of spherical inclusions that appeared to contain mucus‐like material (Figure 1C), as described previously (Rocher et al. 2024). Both exterior and interior surfaces (exo and endo‐pinacoderm) of buds had monociliated cells, whose ciliary beat could be seen by light microscopy (Figure 1D). Both anti‐tubulin labelling and SEM showed that epithelia were monociliate—a single cilium arose from each cell—and that cilia were frequently oriented in a single direction (Figure 1E,F; Rocher et al. 2024).

### 3.2 Ciliary Beat Direction and Particle Tracking in Oscarella Buds

**Figure 2:**
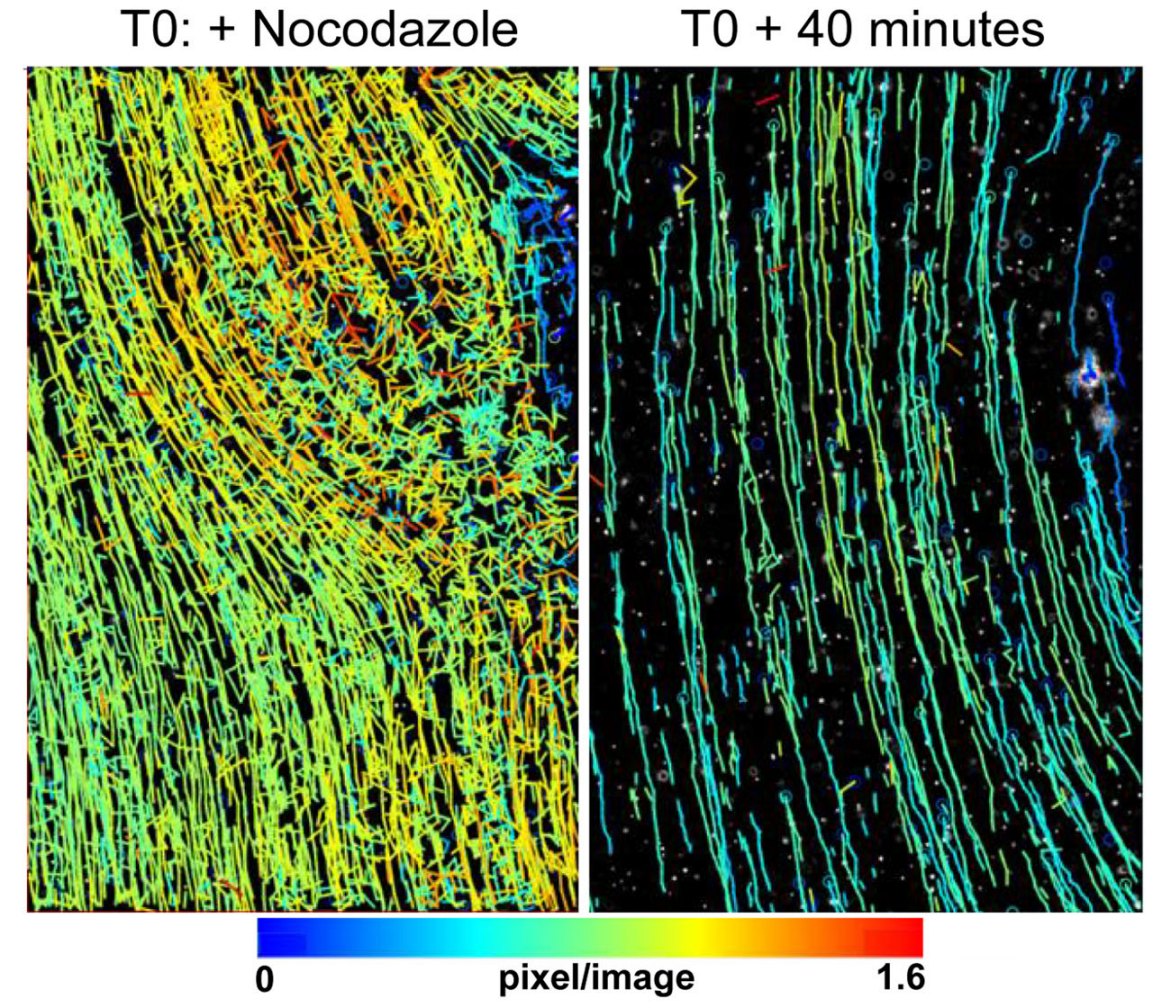
Particle tracking of the movement of fluorescent microspheres on the surface of *Oscarella* sp. Particles tracked on the edge of the outgrowth of a bud show unidirectional movement. Movement is slowed by treatment with nocodazole, which interferes with ciliary function.

Buds that were not attached to the dish (Stage 1 to Stage 3) rotated slowly in the water. On stationary buds, ciliary beat moved small particles in the water across the surface in a single direction. Fluorescent spheres tracked on the surface of buds showed flow was in a common direction and was continuous (Figure 2, left). After 40 min of nocodazole treatment (Figure 2, right) movement of the beads slowed and became discontinuous.

### 3.3 Ciliary Beat Characteristics and Rates in Different Tissues

Different styles of ciliary beat were observed in the various invertebrate tissues studied. Although all cilia beat with the characteristic effective and recovery strokes, some cilia beat continuously at very regular intervals while others beat somewhat irregularly, appearing to stutter, stopping momentarily, before beating again. While all three styles of beat were seen on the buds of *Oscarella* sp., most cilia on the buds beat continuously at regular intervals (Movie 1: S2).

Regular beating was typical of cilia on the clam gill and brachiopod lophophore. Stuttering beats were more typical of the cilia around the rim of the body of *Trichoplax H2*, but were also seen in some of the cilia on the brachiopod lophophore, and irregular and erratic beats were typical on the anemone tentacle and demosponge apopyle cells (Movies 2 and 3: S3 and S4). Nonmotile cilia were also seen on all tissues (Movies 1 and 3: S2 and S4). These cilia were either straight (fully erect) or bent, and did not beat. Nonmotile cilia were very common on the tentacle of *N. vectensis* and on some areas of the lophophore of *T. transversa* (Movie 3: S4).

**Figure 3:**
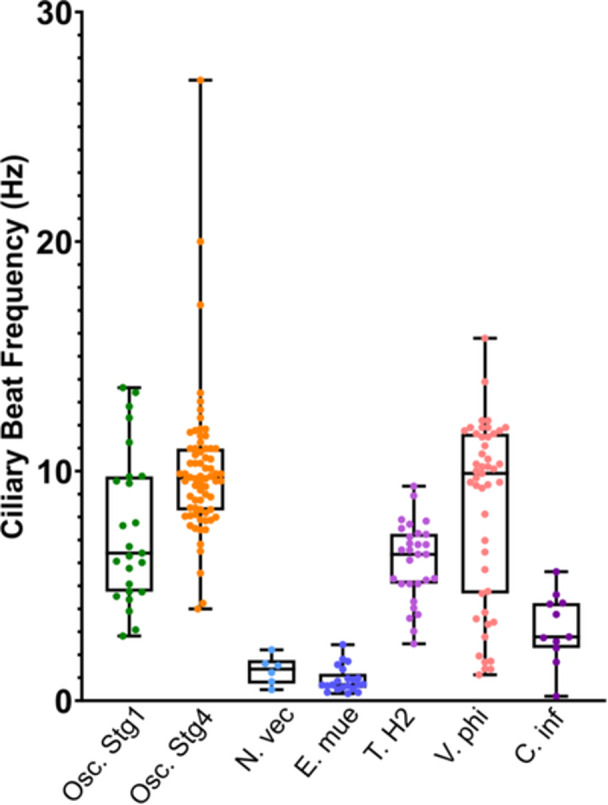
Comparison of ciliary beat frequency across different species. From left to right: *Oscarella* sp. Stage 1, *Oscarella* sp. Stage 4, *Nematostella vectensis, Ephydatia muelleri, Trichoplax* H2, *Venerupis philippinarum, Corella inflata*. The line across each box represents the mean of each data set, while the boundaries of the box represent the lower and upper quartiles. The whiskers represent the maximum and minimum values.

There were differences in ciliary beat rates among the species studied in the lab (Figures 3 and Figure S1). The lowest beat rates were those of *E. muelleri* (0.92 ± 0.56 Hz), *N. vectensis* (1.32 ± 0.62 Hz), and *C. inflata* (3.16 ± 1.52) while the highest were those of *V. philippinarum* (8.20 ± 3.92 Hz) and *Oscarella* sp. Stage 4 (9.99 ± 3.14 Hz) (Table 2). The mean CBFs of buds at Stages 1 and 4 were significantly different (Tables 2 and 3). Cilia from Stage 4 buds beat at a faster rate than cilia from Stage 1 buds (*p* < 0.001, Figure 3, Tables 2 and 3). The CBF of Stage 1 buds was not significantly different from those of *Trichoplax H2* and *V. philippinarum*. The CBF of Stage 4 buds was not significantly different from that of *V. philippinarum*. The CBF of *N. vectensis* was not significantly different from those of apopyle cilia of *E. muelleri* and branchial basket cilia of *C. inflata* (Tables 2 and 3). *T. transversa* was not included in the statistical analysis due to its small sample size.

**Table 2:** Mean ciliary beat rate of the different animals studied. Ciliary beat frequency (CBF) ± standard deviation (SD), minimum and maximum CBF. Mean length (±SD) of the cilia was recorded where possible. NM, not measured.

Species	Mean CBF (Hz)	SD	Min	Max	Mean cilia length (µm)	SD
*Oscarella* sp. Stage 1	7.52	3.23	2.82	13.64	13.04	1.15
*Oscarella* sp. Stage 4	9.99	3.14	4.00	27.03	14.61	0.72
*Nematostella vectensis*	1.32	0.56	0.48	2.22	15.08	1.29
*Ephydatia muelleri*	0.92	0.54	0.31	2.44	8.48	0.24
*Trichoplax H2*	6.06	1.68	2.48	9.35	10.38	0.78
*Venerupis philippinarum*	8.20	3.88	1.13	15.79	14.69	0.35
*Terebratalia transversa*	9.78	1.97	2.44	14.04	18.13	1.86
*Corella inflata*	3.16	1.45	0.20	5.62	NM	

**Table 3:** Results of the ANOVA comparing mean ciliary beat frequencies of *Oscarella* sp., *Nematostella vectensis, Venerupis philippinarum, Ephydatia muelleri, Corella inflata*, and *Trichoplax H2* using a Tukey–Kramer HSD test.

Species compared	*Q* Stat	*p* value
*Oscarella* sp. *Stage 1* vs. *Oscarella* sp. *Stage 4*	5.36	0.0046
*Oscarella* sp. *Stage 1* vs. *N. vectensis*	6.917	< 0.0001
*Oscarella* sp. *Stage 1* vs. *E. muelleri*	11.3	< 0.0001
*Oscarella* sp. *Stage 1* vs.*T. H2*	2.718	0.537
*Oscarella* sp. *Stage 1* vs. *V. philippinarum*	1.783	0.9122
*Oscarella* sp. *Stage 1* vs. *C. inflata*	6.116	0.0006
*Oscarella* sp. *Stage 4* vs. *N. vectensis*	10.33	< 0.0001
*Oscarella* sp. *Stage 4* vs. *E. muelleri*	18.47	< 0.0001
*Oscarella* sp. *Stage 4* vs. *T. H2*	9.015	< 0.0001
*Oscarella* sp. *Stage 4* vs. *V. philippinarum*	4.282	0.0542
*Oscarella* sp. *Stage 4* vs. *C. inflata*	10.67	< 0.0001
*N. vectensis* vs. *E. muelleri*	0.434	> 0.9999
*N. vectensis* vs.*T. H2*	5.358	0.0046
*N. vectensis* vs. *V. philippinarum*	8.272	< 0.0001
*N. vectensis* vs. *C. inflata*	1.836	0.8989
*E. muelleri* vs. *T. H2*	9.087	< 0.0001
*E. muelleri* vs. *V. philippinarum*	14.43	< 0.0001
*E. muelleri* vs. *C. inflata*	3.044	0.3846
*T. H2* vs. *V. philippinarum*	5.011	0.0109
*T. H2* vs. *C. inflata*	4.154	0.0695
*V. philippinarum* vs. *C. inflata*	7.925	< 0.0001

### 3.4 Scanning Electron Microscopy of the Cilia

**Figure 4:**
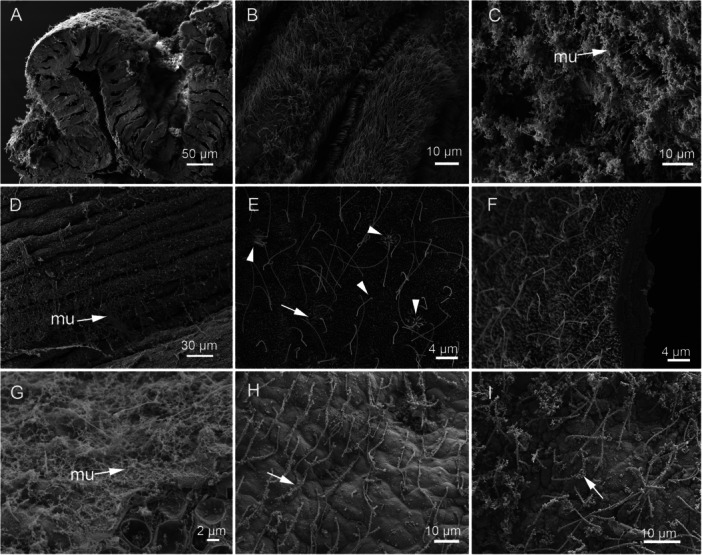
Cilia from invertebrate epithelia and *Oscarella* sp. viewed by SEM. (A) A fracture showing an overview of the gill of *Venerupis philippinarum*. (B) *V. philippinarum* gill at higher magnification fixed without ruthenium red. (C) *V. philippinarum* gill fixed with ruthenium red (mu, mucus). (D) The body wall of the anemone *Nematostella vectensis* fixed with ruthenium red and showing sheets of mucus on the surface (mu, mucus). (E) *N. vectensis* tentacle fixed without ruthenium red. Arrowheads indicate hair cell rosettes around cilia. Arrow indicates a stereocilium (cnidocil). (F) *N. vectensis* tentacle, fixed with ruthenium red, showing the absence of mucus. (G) The surface of a Stage 4 bud of *Oscarella* sp. fixed with ruthenium red shows mucus on the surface (mu, mucus). (H) *Oscarella* sp. Stage 4 fixed without ruthenium red. (arrow, cilium with mucus). (I) *Oscarella* sp. Stage 1 fixed with ruthenium red. Cilia are not oriented in a single direction as in (H), but they are associated with mucus (arrow).

Ruthenium red staining showed mucus on the cilia and surrounding tissues in the gills of *V. philippinarum* and the body surface of *N. vectensis* (Figure 4A–D). In some areas of these two species, the mucus was very densely clumped (Figure 4C,D). Notably, however, the tentacles of *N. vectensis* showed hardly any mucus associated with cilia (Figure 4E,F), even when fixed with ruthenium red (Figure 4F). Whereas cilia on the clam gill were dense, those on the tentacle of *N. vectensis* were sparse. Some cilia were longer than others, ranging in length between 10 and 25 µm (Table 2), and intermittent stereocilia with surrounding microvilli (Watson et al. 2009) were seen (Figure 4E). Most surfaces of the buds that were fixed with ruthenium red were coated with mucus (Figure 4G, I); only a few individual strands of mucus were found on those fixed in the absence of ruthenium red (Figure 4H).

### 3.5 Meta‐Analysis of Ciliary Beat Rates

**Figure 5:**
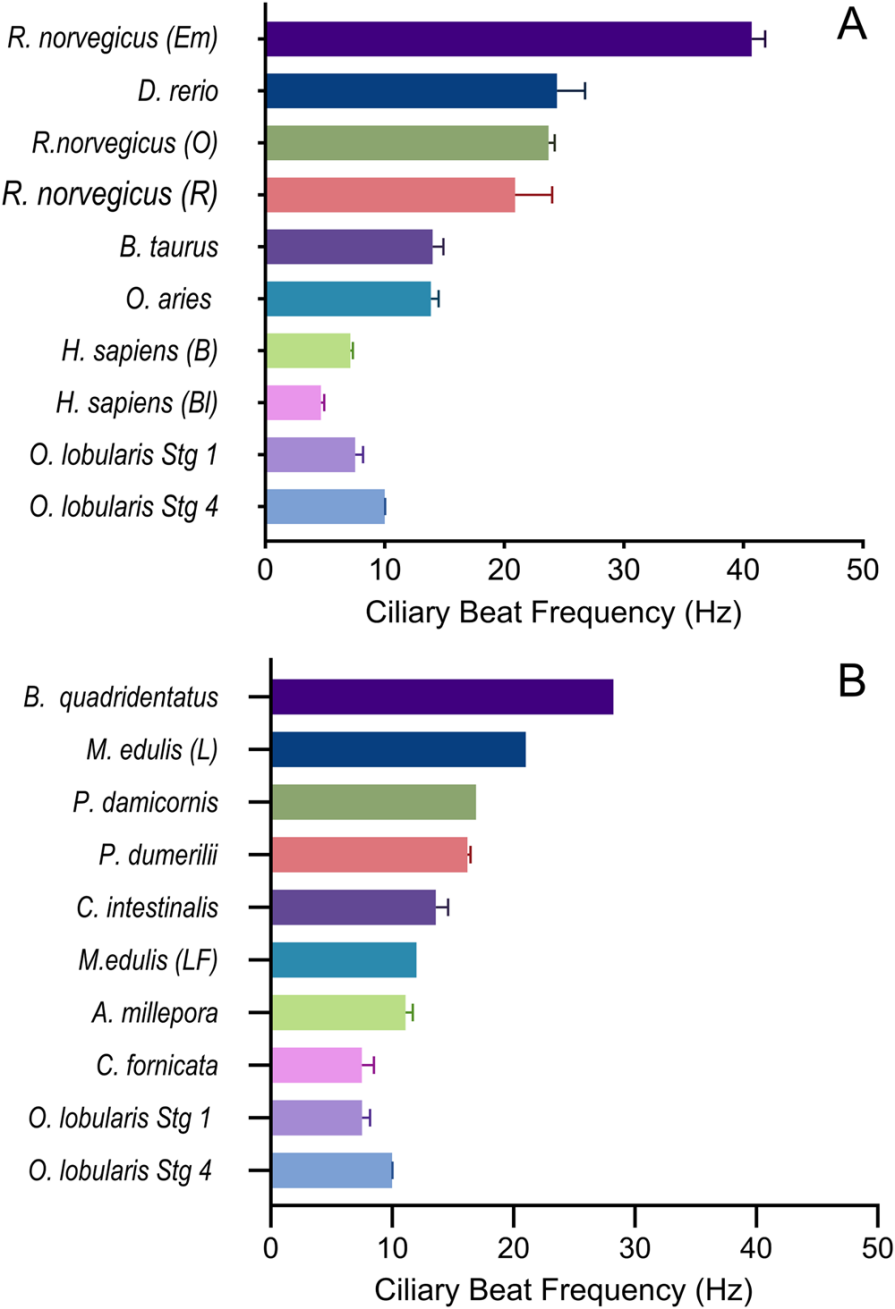
Meta‐analysis of ciliary beat frequencies (CBFs) from the primary literature. (A) CBFs of vertebrates compared with those of *Oscarella* sp. Stages 1 and 4. From top to bottom: rat ependymal cilia (O'Callaghan et al. 1999); zebrafish nasal cilia (Reiten et al. 2017); rat oviductal cilia (Halbert et al. 1989); rat respiratory cilia (O'Callaghan et al. 1999); cow bronchi cilia (Joki and Saano 1994); lamb tracheal cilia (Kelly et al. 2021); human bronchi cilia (Clary‐Meinesz et al. 1997); human bronchiole cilia (Clary‐Meinesz et al. 1997); *Oscarella* sp. Stage 1 and *Oscarella* sp. Stage 4. Error bars indicate standard error. (B) CBFs of invertebrates compared with those of *Oscarella* sp. Stages 1 and 4. From top to bottom: rotifer oral cilia (Doyle et al. 2006); mussel lateral cilia (Riisgård et al. 1996); coral epithelial cilia (Shapiro et al. 2014); annelid larvae (Conzelmann et al. 2011); *Ciona* lateral cilia; mussel latero‐frontal cilia (Riisgård et al. 1996); reef coral larvae (Poon et al. 2022); marine gastropod larva (Penniman et al. 2013); *Oscarella* sp. Stage 1; and *Oscarella* sp. Stage 4. Error bars indicate SE.

The CBFs of vertebrate and invertebrate species from the primary literature were compared with those of *Oscarella* sp. (Figure 5). There were insufficient data points reported in the different studies found for robust statistical analysis; however the CBFs of *Oscarella* sp. buds were generally lower than those of all the vertebrates except for cilia of human bronchi and bronchioles. The CBF of Stage 1 buds of *Oscarella* sp. (7.52 Hz) was similar to that of cilia on larvae of *Crepidula fornicata* (Mollusca) (7.5 Hz). The CBF of Stage 4 buds (9.99 Hz) was closest to those of larval cilia of the coral (Cnidaria) *Acropora millepora* (11.1 Hz) and the latero‐frontal gill cilia of *Mytilus edulis* (Mollusca) (12 Hz).

## 4 Discussion

Cilia are such common organelles on all metazoan epithelia that it is surprising they are absent on all adult sponges except for those in the class Homoscleromorpha. While other studies have documented the presence of single cilia on epithelia of homoscleromorph sponges (Ereskovsky et al. 2007; Borchiellini et al. 2021; Renard, Rocher, et al. 2021; Rocher et al. 2024), none have yet examined what their function might be nor questioned why other sponges lack cilia on their pinacoderms. Here we asked if the function of cilia on homoscleromorph sponge epithelia could be discerned by studying their beating activity, particle tracking, and aspects of their morphology. We also carried out a meta‐analysis to compare rates of ciliary beat in *Oscarella* sp. with those of other metazoan epithelia reported in the literature. We found that, although some cilia were clearly stationary (i.e., nonmotile), most cilia on buds were motile and that beat rates were in the range of those of other ciliary mucus movers. Mucus, as detected by fixing tissues with ruthenium red, was also clearly associated with the cilia.

### 4.1 Morphology and Orientation of Cilia on Buds of *Oscarella* sp

The buds that come off the adult sponges of *Oscarella* sp. either naturally or in vitro, are not only fascinating as a method of asexual reproduction, but an extremely practical model to study (Renard, Rocher et al. 2021; Rocher et al. 2024). They are small enough to fit under a coverslip and yet they contain all the functioning components of a young sponge. Being able to film ciliary beat live, and with the addition of fluorescent microspheres, allowed us to determine that ciliary beat was organized and unidirectional. It is possible that their beat was entrained, although as cells were monociliate the ciliary coat was sparse and no metachronal waves were seen by high‐speed imaging. The fact that the movement of particles was driven by the cilia was confirmed by treatment of buds with nocodazole, which substantially reduced the movement of beads across the bud surface.

### 4.2 Rates of Ciliary Beat as a Test of Function

The beat frequency of cilia is determined by many factors, including viscosity of the surrounding medium (and therefore temperature), which fluid is being transported, length of cilia, and availability of ATP and Ca^2+^ ions (Aiello 1974; Gilpin et al. 2020). It is therefore likely that cilia performing similar functions in a similar environment will have similar beat frequencies. This idea is supported by the fact that cilia with a similar function, such as those of the gill of *V. philippinarum* which moves mucus, has a higher beat frequency than cilia of the anemone tentacle and sponge apopyle which are slow and beat infrequently suggesting a sensory role (Watson et al. 2009; Nickel 2010; Bezares‐Calderón et al. 2020).

We found that the CBF of Stage 1 buds of *Oscarella* was significantly slower than those of Stage 4, which were more similar to those of the clam gill. There was no significant difference between the CBFs of Stage 1 buds and *Trichoplax H2*. Some of the cilia of *Trichoplax adhaerens* are used to move the animal along surfaces coated with mucus secreted from gland cells in its epithelium, but others at the margin of the animal may have a sensory function (Mayorova et al. 2019). The cilia we measured were largely at the margin where their beat could be clearly seen. As such these may have had different functions, sensory and mucus movement, and images showing mucus where those cilia are on the surface of *T. adhaerens* (Smith et al. 2021) suggests that their CBF may be comparable to those of other species which use their cilia for mucus transport.

Altogether, the lack of a significant difference between the CBF of *Oscarella* sp. buds at Stage 4 and cilia on the gills of *V. philippinarum* further supports the hypothesis that *Oscarella* sp. and other homoscleromorph species use cilia for mucus‐driven particle transport. It is well‐known that the gills of clams have goblet cells that secrete mucus and that the mucus is used for transporting trapped particles to the labial palps (Davies and Hawkins 1998), and accordingly we found cilia on the gill of *V. philippinarum* were covered in a heavy coat of mucus.

The reason for the significantly different CBFs of the two stages of *Oscarella* sp. buds remains unclear. It is possible that the cilia change their function over the course of the bud's development. As budding is a natural occurrence, we can infer that the cilia allow the new tiny buds to disperse when they are first released. In that case, the slower beat rate may be sufficient to keep the bud in the water column as currents move it. On the other hand, it could be that cilia from very young buds simply cannot beat as quickly and that the more mature buds have more resources such as ATP and Ca^2+^ to devote to ciliary beating. Another possibility is that beating of exopinacocyte cilia is somehow involved in particle filtering. If so, then the different beating rates observed here between young buds and settled juveniles may partly explain the differences in the efficiency of particle capture reported previously (Rocher et al. 2024). Taken together, these results suggest that the beating cilia on *Oscarella* sp. epithelia may be for motility in the early bud stage, but they are more likely to function in movement of mucus as buds mature and in the sessile attached juveniles and adults.

In many metazoans cilia are most often used for moving mucus and keeping surfaces clean, and although sponges do clean their surfaces by moving mucus across the epithelium as recently shown for Caribbean sponges (Kornder et al. 2022), most sponges clearly do this without cilia, as none, except for homoscleromorphs, has cilia on their outer surfaces. Kornder et al. (2022) studied the demosponge *Aplysina aerophoba* using time‐lapse photography and showed that particles move across the surface of the sponge in strands of mucus, to collect in clumps which are periodically shed from the surface by a single coordinated contraction (a sneeze). The rate of particle movement in mucus over *A. aerophoba* was 2 µm s^−1^, whereas particle movement in mucus on different ciliary mucus feeders ranges from 70 µm s^−1^ in pelagic tunicates to up to 400 µm s^−1^ in various clam gills (Ward et al. 1993; Luis Acuña et al. 1996). Clearly mucus transport in sponges that lack cilia is exceedingly slow, but nevertheless is effective. How the transport occurs is still unknown, but given the rate is similar to cell crawling, it may be a type of pseudopodial ‘walking’ of material over the cell's surface. Future studies could compare rates of mucus transport across demosponges and homoscleromorph sponges.

### 4.3 Meta‐Analysis and Comparison of CBF Across Metazoans

The meta‐analysis of CBFs across invertebrates and vertebrates provides useful comparisons for the ciliary function of epithelia in Homoscleromorpha, with the caveat that experimental conditions such as temperature varied between studies, and entire data sets were not always available, preventing statistical comparisons. Most of the CBFs obtained from mammals were significantly higher than those obtained from the buds of *Oscarella* sp. These ranged in function from circulating cerebrospinal fluid, transporting mucus, and transporting eggs, but they were recorded at much higher temperatures, ranging from 28°C to 37°C, than the invertebrate tissues studied. As temperature is known to impact CBF and the viscosity of mucus, direct comparison with this data is difficult (Gilpin et al. 2020). Some vertebrate CBFs were recorded at lower temperatures (17°C–22°C), which would have been similar to our experimental conditions. Mouse small airway cilia from this temperature range had a mean beat frequency of 12.45 Hz, which is faster but close to that of Stage 4 buds of *Oscarella* sp. (9.99 Hz), though it should be noted that these are multiciliated cells and the epithelial cells of Homoscleromorpha are monociliated (Delmotte and Sanderson 2006). Furthermore, the CBF of human bronchial cilia measured at 22°C was 7.11 Hz, which is very close to that of Stage 1 buds of *Oscarella* sp. (7.52 Hz) (Clary‐Meinesz et al. 1997). As mammalian airways clear debris by mucociliary action, the similarity of these values to our own results further supports the hypothesis that the cilia of *Oscarella* sp. transport mucus across its epithelial surface.

Studies of cilia used for movement of water such as the feeding cilia of rotifers (28.23 Hz), vortex creating cilia of coral (16.9 Hz), and current‐creating lateral cilia of mussels (21 Hz) and *Ciona* (13.6 Hz) provided CBFs considerably higher than those of Stage 1 and 4 buds (Riisgård et al. 1996; Petersen et al. 1999; Doyle et al. 2006; Shapiro et al. 2014). These studies were conducted at a similar temperature range to our own. The much higher CBFs of these current‐producing cilia make it seem unlikely that *Oscarella* species use cilia to enhance flow towards and into the incurrent canals, or to draw particles captured in the mucus into the canals for muco‐ciliary feeding. The closest CBFs to Stage 4 buds of *Oscarella* sp. were latero‐frontal cilia of mussels (12.0 Hz), used for particle capture, and the locomotory cilia of coral larvae (11.1 Hz) (Riisgård et al. 1996; Poon et al. 2022). The CBF of *Crepidula fornicata* larvae (about 7.5 Hz), also used for locomotion, was very similar to that of Stage 1 buds (Penniman et al. 2013), suggesting therefore that the function of cilia on the surface of very young buds (Stage 1–3) may be for motility and dispersal as discussed above, but for older buds that are sessile (Stage 4), and for adult sponges, the cilia are not used for locomotion.

## 5 Conclusions

Our study has highlighted a number of important features of cilia on the epithelia of homoscleromorph sponges, and what we reveal brings up some interesting considerations. First, our observations firmly indicate that these organelles move in the manner of cilia, driving flow along the epithelium, rather than as flagella, drawing flow up and away from the epithelium. Use of the term flagella for these organelles in homoscleromorphs, as seen in some previous work (Gazave et al. 2010; Ereskovsky et al. 2014) can be misleading for understanding their function, and so our study helps to clarify the function of these organelles for future comparisons.

**Figure 6:**
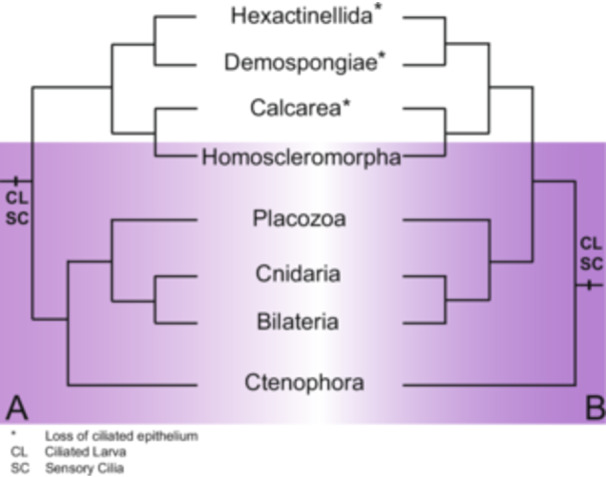
Evolutionary hypotheses describing the loss of the ciliated epithelium in three classes of sponge. (A) Evolutionary hypothesis which places Porifera as the sister group to the rest of Metazoa. (B) Evolutionary hypothesis which places Ctenophora as the sister group to the rest of Metazoa. Groups which possess a ciliated epithelium are highlighted in purple. Asterisks denote groups that lack a ciliated epithelium. cl: ciliated larva. sc: sensory cilia.

Second, while our data strongly suggest that cilia on homoscleromorphs are used for mucus‐driven particle transport, it remains unclear why only one class of sponge has ciliated epithelia. Sponges of classes Demospongiae, Calcarea, and Hexactinellida can clean their surfaces and feed without the use of a ciliated epithelium, so it is unknown why class Homoscleromorpha has invested in this energetically costly structure while other sponges have not. As almost all sponge larvae are ciliated (Woollacott and Pinto 1995; Maldonado and Bergquist 2002), and as cilia can be found on the lining of the oscula of other classes of sponges (Nickel 2010; Nickel et al. 2011; Ludeman et al. 2014) the ancestral condition of Porifera must have included a ciliated pinacoderm (external and internal epithelium), which has been lost by the other three classes (Figure 6). Our data intriguingly suggest that by possessing cilia, homoscleromorph epithelia are more similar to the epithelia of other metazoans, not only at the cellular and molecular levels (Renard, Le Bivic, et al. 2021) but also at a physiological level. Further genomic studies could shed light on this issue.

## Author Contributions

Conceptualization: Sally P. Leys, Emmanuelle Renard, Carole Borchiellini, Andre Le Bivic. Acquisition of video/image‐data: Sally P. Leys, Veronica L. Price, Andrea Pasini, Elsa Bazellieres, Carole Borchiellini, Caroline Rocher, and Amelie Vernale. Video data processing: Veronica L. Price, Anudi Nanayakkara. Analysis of data: Veronica L. Price, Anudi Nanayakkara, Elsa Bazellieres, and Sally P. Leys. Supervision and project administration: Sally P. Leys, Andre Le Bivic, Elsa Bazellieres, Emmanuelle Renard, Carole Borchiellini. Writing – original draft: Veronica L. Price, Sally P. Leys. Writing – review and editing: All authors.

## Ethics Statement

The authors have nothing to report.

## Conflicts of Interest

The authors declare no conflicts of interest.

## Supporting information


**Figure S1:** Histograms showing the distribution of ciliary beat frequencies (CBF) in the organisms studied.

**Movie 1:** Oscarella smaller.

**Movie 2:** Irregular Beats.

**Movie 3:** Non‐motile Cilia.

## Data Availability

Data is available at the University of Alberta Education and Research Archive https://ualberta.scholaris.ca/items/792f0804-ae69-4364-8a17-63c4f1d16bf2.
