# Supplementary material for: Why Homoscleromorph Sponges Have Ciliated Epithelia: Evidence for an Ancestral Role in Mucociliary Driven Particle Flux
Source: J Exp Zool B Mol Dev Evol. 2025 Aug 22;344(8):505–16. doi: 10.1002/jez.b.23324 (PMC12626907; doi:10.1002/jez.b.23324)
Supplement: Supplementary file 1 — Figure S1: Histograms showing the distribution of ciliary beat frequencies (CBF) in the organisms studied. [file JEZ-344-505-s004.pdf]

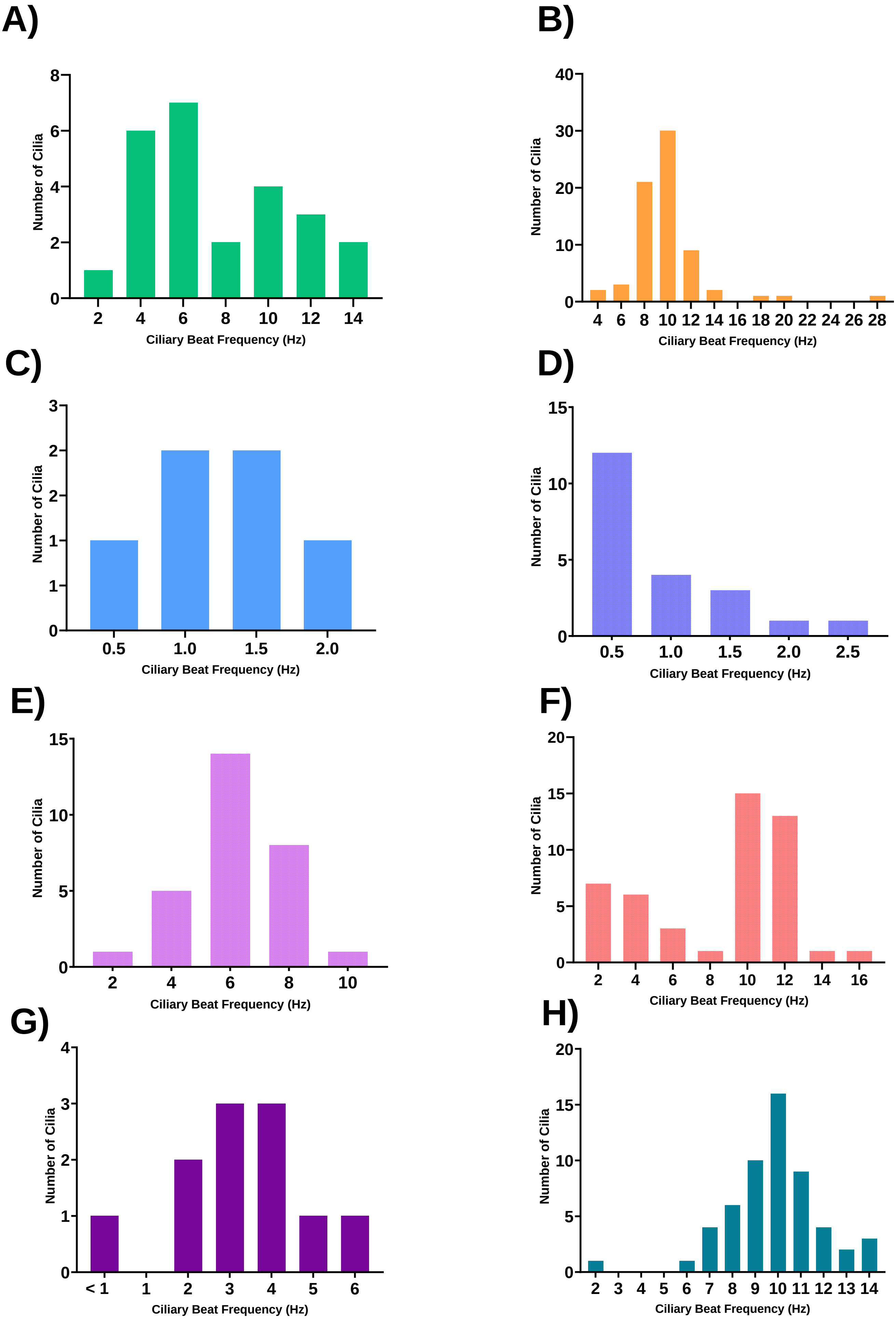
A)
Number of Cilia
0 2 4 6 8
2 4 6 8 10 12 14
Ciliary Beat Frequency (Hz)
B)
Number of Cilia
0 10 20 30 40
4 6 8 10 12 14 16 18 20 22 24 26 28
Ciliary Beat Frequency (Hz)
C)
Number of Cilia
0 1 1 2 2 3
0.5 1.0 1.5 2.0
Ciliary Beat Frequency (Hz)
D)
Number of Cilia
0 5 10 15
0.5 1.0 1.5 2.0 2.5
Ciliary Beat Frequency (Hz)
E)
Number of Cilia
0 5 10 15
2 4 6 8 10
Ciliary Beat Frequency (Hz)
F)
Number of Cilia
0 5 10 15 20
2 4 6 8 10 12 14 16
Ciliary Beat Frequency (Hz)
G)
Number of Cilia
0 1 2 3 4
< 1 1 2 3 4 5 6
Ciliary Beat Frequency (Hz)
H)
Number of Cilia
0 5 10 15 20
2 3 4 5 6 7 8 9 10 11 12 13 14
Ciliary Beat Frequency (Hz)

Supplementary Figure 1. Histograms showing the distribution of ciliary beat frequencies (CBF) in the organisms studied. Y-axis represents number of beats counted at each frequency shown on the X-axis. (A) Stage 1 *Oscarella sp*.; beats of 25 cilia were measured on 3 different buds; (B) Stage 4 *Oscarella sp*.; 70 cilia were measured on 3 different buds; (C) *Nematostella vectensis*; 6 cilia were measured on 2 individuals; (D) *Ephydatia muelleri*; 21 cilia were measured on 3 individuals; (E) *Trichoplax H2*; 29 cilia were measured on 3 individuals (F) *Venerupis philippinarum*; 47 cilia were measured on 3 individuals. (G) *Corella inflata*; 11 cilia were measured on 3 individuals; (H) *Terebratalia transversa*; 56 cilia were measured on one individual. *T. transversa* data were not included in the larger analysis as only one individual was available for study.
